# No indications of weight gain associated DNA methylation changes in patients with anorexia nervosa

**DOI:** 10.1038/s41598-025-12592-5

**Published:** 2025-08-07

**Authors:** Luisa Sophie Rajcsanyi, Miriam Kesselmeier, Christopher Schröder, Carolin Schmelting, Triinu Peters, Manuel Föcker, Isabelle Kraft, Jasmin Beygo, Elsa Leitão, Michael Zeschnigk, Johanna Giuranna, Beate Herpertz-Dahlmann, Jochen Seitz, Martina de Zwaan, Wolfgang Herzog, Stefan Ehrlich, Stephan Zipfel, Katrin Giel, Karin Egberts, Roland Burghardt, Bettina Budeus, Johannes Hebebrand, Bernhard Horsthemke, Anke Hinney

**Affiliations:** 1 Section of Molecular Genetics in Mental Disorders, University Hospital Essen, University of Duisburg-Essen, Virchowstr 174, 45147 Essen, Germany; 2 Department of Child and Adolescent Psychiatry, Psychosomatics and Psychotherapy, University Hospital Essen, University of Duisburg-Essen, Essen, Germany; 3 Center for Translational Neuro- and Behavioural Sciences, University Hospital Essen, Essen, Germany; 4 Institute for Sex and Gender-Sensitive Medicine, University Hospital Essen, Essen, Germany; 5 Institute of Medical Statistics, Computer and Data Sciences (IMSID), Jena University Hospital / Friedrich Schiller University Jena, Jena, Germany; 6 Institute of Human Genetics, University Hospital Essen, University of Duisburg-Essen, Essen, Germany; 7 Genome Informatics, Institute of Human Genetics, University Hospital Essen, University of Duisburg-Essen, Essen, Germany; 8 Department of Child and Adolescent Psychiatry, Psychosomatics and Psychotherapy, University Hospital Münster, Münster, Germany; 9 LWL-University Hospital Hamm for Child and Adolescent Psychiatry, Ruhr-University Bochum, Hamm, Germany; 10 Department of Child and Adolescent Psychiatry, Psychosomatics and Psychotherapy, University Hospital of the RWTH Aachen, Aachen, Germany; 11 Department of Psychosomatic Medicine and Psychotherapy, Hannover Medical School, Hannover, Germany; 12 Department of Internal Medicine II, General Internal and Psychosomatic Medicine, University of Heidelberg, Heidelberg, Germany; 13 Translational Developmental Neuroscience Section, Department of Child and Adolescent Psychiatry, University Hospital Carl Gustav Carus Dresden, University of Technology Dresden, Dresden, Germany; 14 Eating Disorders Research and Treatment Center, Department of Child and Adolescent Psychiatry, Faculty of Medicine, TU Dresden, Dresden, Germany; 15 Department of Psychosomatic Medicine and Psychotherapy, Medical University Hospital Tübingen, Tübingen, Germany; 16 Center of Excellence in Eating Disorders KOMET, Tübingen, Germany; 17 German Center for Mental Health (DZPG), Tübingen, Germany; 18 Department of Child and Adolescent Psychiatry, Psychosomatics and Psychotherapy, University of Würzburg, Würzburg, Germany; 19 Department of Child and Adolescent Psychiatry, Reinier Van Arkel, ‘s-Hertogenbosch, The Netherlands; 20 Child and Adolescent Psychiatry Clinic, Oberberg Fachklinik Fasanenkiez Berlin, Berlin, Germany; 21 Genomics and Transcriptomics Facility, University Hospital Essen, Essen, Germany

**Keywords:** DNA methylation, Genetics research

## Abstract

Anorexia nervosa (AN) is a mental disorder marked by a significantly low body weight. Differentially methylated CpG sites have been reported to be involved in body weight regulation. Methylation pattern may change during considerable weight gain by in-patient treatment. Consequently, we aimed to (1) replicate the hypomethylation at the *NR1H3* gene locus (identified in our previous epigenome-wide association study) in independent study groups of 189 female patients with AN and 67 healthy-lean female controls, and (2) identify regions associated with large weight gain associated DNA methylation changes in three patients with AN through whole-genome bisulfite sequencing in CD14^+^ cells. In the replication study, no evidence was observed for hypomethylation at the investigated 15 CpG sites of the *NR1H3* locus. Relying on two analysis tools (camel, metilene) to identify differentially methylated regions (DMRs), subtle methylation differences concordant between both tools were detected only when the usual threshold of camel was lowered. Then, eight regions were selected exemplarily for technical replication with deep bisulfite sequencing in the same three patients with AN. None of the regions could be confirmed. Summarising, we could not confirm hypomethylation at *NR1H3* and could not detect methylation differences in patients with AN between admission and weight gain at discharge.

**Supplementary Information:**

The online version contains supplementary material available at 10.1038/s41598-025-12592-5.

## Introduction

Human body weight is regulated by both genetic and environmental factors^1^. Anorexia nervosa (AN) is a severe, potentially life-threatening mental disorder that typically manifests during late childhood and adolescence^2,3^. Females are approximately ten times more often diagnosed than males with lifetime prevalence rates of 0.8% in females in the US^2^. The diagnosis is based on a significantly low body weight and AN-related psychopathology including weight and shape concerns^4^. Starvation is a core physiological feature of actue AN^5^. Accordingly, the symptomatology results from an age-, duration- and severity-dependent intertwining of primary cognitive and behavioral features with starvation induced somatic, cognitive and behavioral symptoms^5^.

Previously, it has been shown, that caloric restriction alters DNA and histone modifications (acetylation, methylation)^6,7^. For instance, it was shown that individuals who were prenatally exposed to famine (Dutch Hunger Winter in 1944–45) showed six decades later reduced DNA methylation at the imprinted *IGF2* gene locus, when compared with unexposed, same-sex siblings^8^.

For AN, epigenetic studies are still scarce^9,10^. So far, the DNA methylation of a number of candidate genes has been analysed (e.g.^11–19^) and solely few epigenome-wide association studies (EWAS) have been performed^20–24^ (reviewed by ^9,10^). Given that DNA methylation patterns are highly cell-type specific^25,26^, it remains uncertain whether the previously reported DNA methylation changes indeed reflect true alterations due to diseases and/or starvation, respective interventions or whether the detected changes merely reflect differences in the cellular composition of the investigated tissue. All previously published studies investigated methylation patterns in whole blood, DNA from saliva or buccal cells^10^. Moreover, factors like infections^27^, menstrual cycle^28^ and stress^29^ influence the highly dynamic cell composition of leukocytes. Thus, sampling at different time points might induce DNA methylation differences^30^.

Previously, we examined DNA methylation derived from whole blood of 47 females with AN, as well as two control groups of 47 lean females without AN and 100 population-based females^21^. We applied two reference-free methods (FastLMM-EWASher, RefFreeEWAS) to account for different cell type compositions and searched for consensus CpG sites identified by both methods. In this study, 51 consensus sites were identified in AN versus lean and 81 in AN versus population-based comparisons. These sites had not previously been reported in AN methylation analyses. By comparing the consensus sites with the literature and a genome-wide association study meta-analysis, a single nucleotide polymorphism (rs923768) in *CSGALNACT1* was identified to be nominally associated with AN, while at gene level, hypermethylation at *TNXB* in patients with AN compared to controls was confirmed^21^. Our analysis of AN versus lean controls further revealed evidence for a relevant locus at *NR1H3*^21^. However, the methylation was opposite to the one previously reported^20^. Collectively, we confirmed genes like *TNXB* described to comprise differentially methylated sites, and highlighted further sites that might be specifically involved in AN^21^.

In accordance, Steiger and colleagues conducted a genome-wide DNA methylation analysis in whole blood of 75 female patients with AN, 31 females with remitted AN and 41 females with no diagnosed eating disorder. Hereby, methylation levels and directions differed between individuals suffering from acute AN and patients with remitted AN indicating reversible epigenetic alterations^22^. Likewise, a larger study demonstrated reversible methylation patterns in individuals with AN^23^. An investigation of whole blood samples of seven monozygotic AN-discordant twin pairs revealed nine gene-linked differentially methylated CpG sites. When validating these with a rank regression model as well as a beta regression, two and six CpG sites associated with genes remained significant, respectively. The correlated genes were mainly linked to metabolic and psychological traits^31^.

Within the present study, we aim to investigate methylation changes in patients with AN in more detail. First, as we identified a hypomethylation at *NR1H3* within our EWAS approach^21^ in contrast to the previously reported hypermethylation^20,23^, we aimed to replicate the hypomethylation at *NR1H3* in 198 female patients with AN and 87 healthy-lean female controls using whole blood samples. Secondly, we aim to validate the hypothesis that a weight gain achieved in in-patient treatment considerably alters the methylome across a wide range of cell and tissue types. Therefore and to further address the above-mentioned limitations of DNA methylation studies, we aimed to identify *de novo* and genome-wide differentially methylated regions (DMRs) before and after weight gain in three patients with AN by performing whole-genome bisulfite sequencing (WGBS) of DNA isolated from the CD14^+^ cell population. As patients with AN tend to gain a considerable amount of weight during short-term interventions, we assume that if a link between DNA methylation and body weight regulation exists, we should be able to observe this link when analysing these patients. Subsequently, we conducted a technical replication of the detected *de novo* DMRs with deep bisulfite sequencing in these same three patients. This study design can reduce the amount of false positives resulting from variation in DNA sequence and cell type composition, which are the major confounding factors in DNA methylation studies^32^.

## Results

### Replication analyses of the *NR1H3* gene locus

In the independent study subgroup, we identified at six CpG sites nominal differences in the mean methylation values between females with AN (n = 189) and healthy-lean controls (n = 67; see Table 1; CpG sites #1, #2, #10, #11, #13 and #14). Among them, two showed a hypomethylation in females with AN (#1 and #2), while four were hypermethylated (#10, #11, #13 and #14). Yet, the methylation differences were small (see Table 1). These results are consistent with those from the dependent study subgroup, i.e. the complete sample. One of the identified CpG sites (#11) was also included in our previous study^21^ that accounted for the underlying cell type distributions^33,34^. There, no evidence for methylation differences could be observed for #11. Note, in the previous study, we observed the hypomethylation at *NR1H3* on the gene level, but not at CpG site level.

**Table 1:** DNA methylation differences at the *NR1H3* gene locus between females with and healthy-lean female controls.

CpG site	EWAS data	Replication study data	
		Independent sample	Non-independent sample
cg1		− 0.023 (− 0.044, − 0.002)	− 0.027 (− 0.046, − 0.008)
cg2		− 0.031 (− 0.053, − 0.009)	− 0.035 (− 0.055, − 0.016)
cg3		− 0.009 (− 0.030, 0.013)	− 0.012 (− 0.031, 0.007)
cg4		− 0.002 (− 0.024, 0.020)	− 0.006 (− 0.026, 0.013)
cg5		− 0.004 (− 0.025, 0.018)	− 0.007 (− 0.026, 0.012)
cg6		0.008 (− 0.014, 0.031)	0.005 (− 0.016, 0.025)
cg7 (cg03732020)	− 0.002 (− 0.015, 0.011)	0.009 (− 0.014, 0.032)	0.005 (− 0.016, 0.025)
cg8		0.011 (− 0.010, 0.031)	0.007 (− 0.011, 0.026)
cg9		0.021 (− 0.001, 0.043)	0.019 (0.000, 0.039)
cg10		0.021 (0.002, 0.041)	0.020 (0.002, 0.037)
cg11 (cg09548275)	0.001 (− 0.016, 0.017)	0.022 (0.001, 0.043)	0.022 (0.003, 0.040)
cg12 (cg00347584)	− 0.002 (− 0.015, 0.012)	0.006 (− 0.005, 0.016)	0.003 (− 0.007, 0.012)
cg13		0.009 (0.002, 0.017)	0.008 (0.001, 0.015)
cg14		0.015 (0.003, 0.028)	0.014 (0.002, 0.025)
cg15		0.003 (− 0.002, 0.008)	0.003 (− 0.002, 0.008)

Estimated methylation differences with the corresponding 95% confidence intervals from the robust linear regression modelling are provided. Data of the previously published high-throughput EWAS analysis (^21^) and of the present replication study are presented. Females already analysed in the EWAS study were excluded from the independent study group and were consequently included in the non-independent samples. If the 95% confidence interval does not include 0, the difference is statistically significant.

### Identification of putatively weight gain associated DMRs

Within our exploratory study in three female patients with AN receiving in-patient treatment, in which all females gained considerably weight (see Table 2), we did not observe evidence for DMRs between admission and weight gain based on camel applying the proposed thresholds. If we lowered the thresholds and increased the stringency, we identified 35 DMRs (see Supplementary Table 4). Relying on metilene, we observed 30 DMRs (see Supplementary Table 5). Thirteen DMRs were detected with both tools (see Supplemenatry Tables 4 and 5). With regard to our DMR identification and based on the publication by Ziller et al.^35^, we estimated that we reached a true positive rate of about 80% and a false positive rate of about 20%.

**Table 2:** Phenotypic characteristics of the three female patients with AN used for the identification for DMRs.

Characteristic	Patient #1	Patient #2	Patient #3
Age, in years	16	16	15
Treatment duration, in days	71	100	100
Height, in cm			
At admission	154.5	173.8	159.2
At discharge	154.5	173.8	159.2
Weight, in kg			
At admission	34.0	46.1	38.3
At discharge	41.8	53.0	44.6
BMI, in kg/m^2^			
At admission	14.2	15.3	15.1
At discharge	17.5	17.6	17.6

BMI, body mass index, DMRs, differentially methylated regions.

**Fig. 1:**
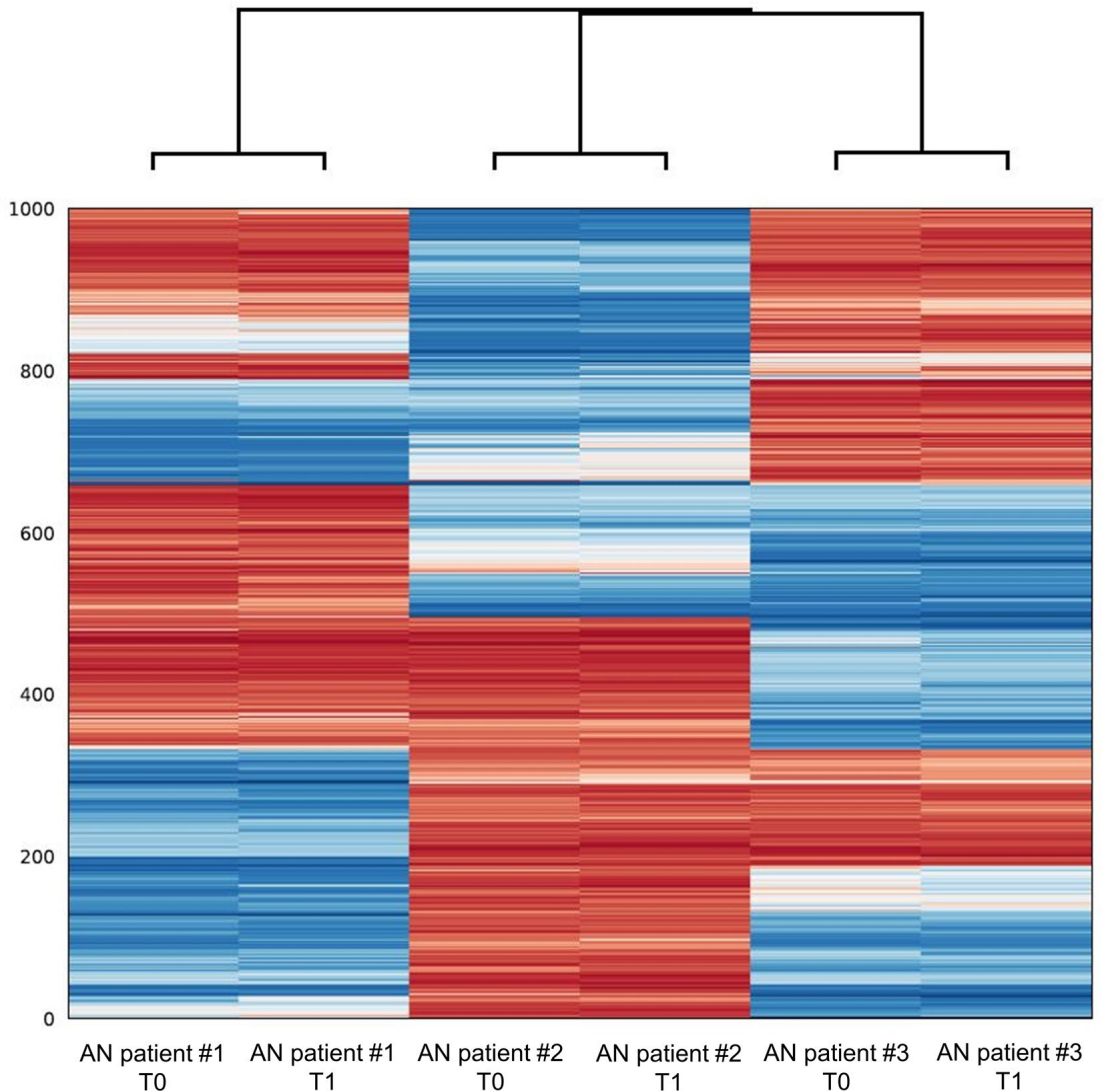
Cluster analysis of the 1,000 most variable CpG sites. The obtained methylomes of pre- (T0) and post-treatment-induced weight gain (T1) from three female patient with anorexia nervosa (AN) are shown.

The principal component analysis (PCA) did not reveal a stringent grouping of the samples according to pre- and post-intervention (see Supplementary Fig. 1). Similarly, when performing a cluster analysis of the 1,000 most variable CpG sites, each individual showed distinct methylation patterns (see Fig. 1). These detected inter-individual methylation differences most likely reflect the different genetic backgrounds.

### Technical replication of WGBS-resultant targets

**Fig. 2:**
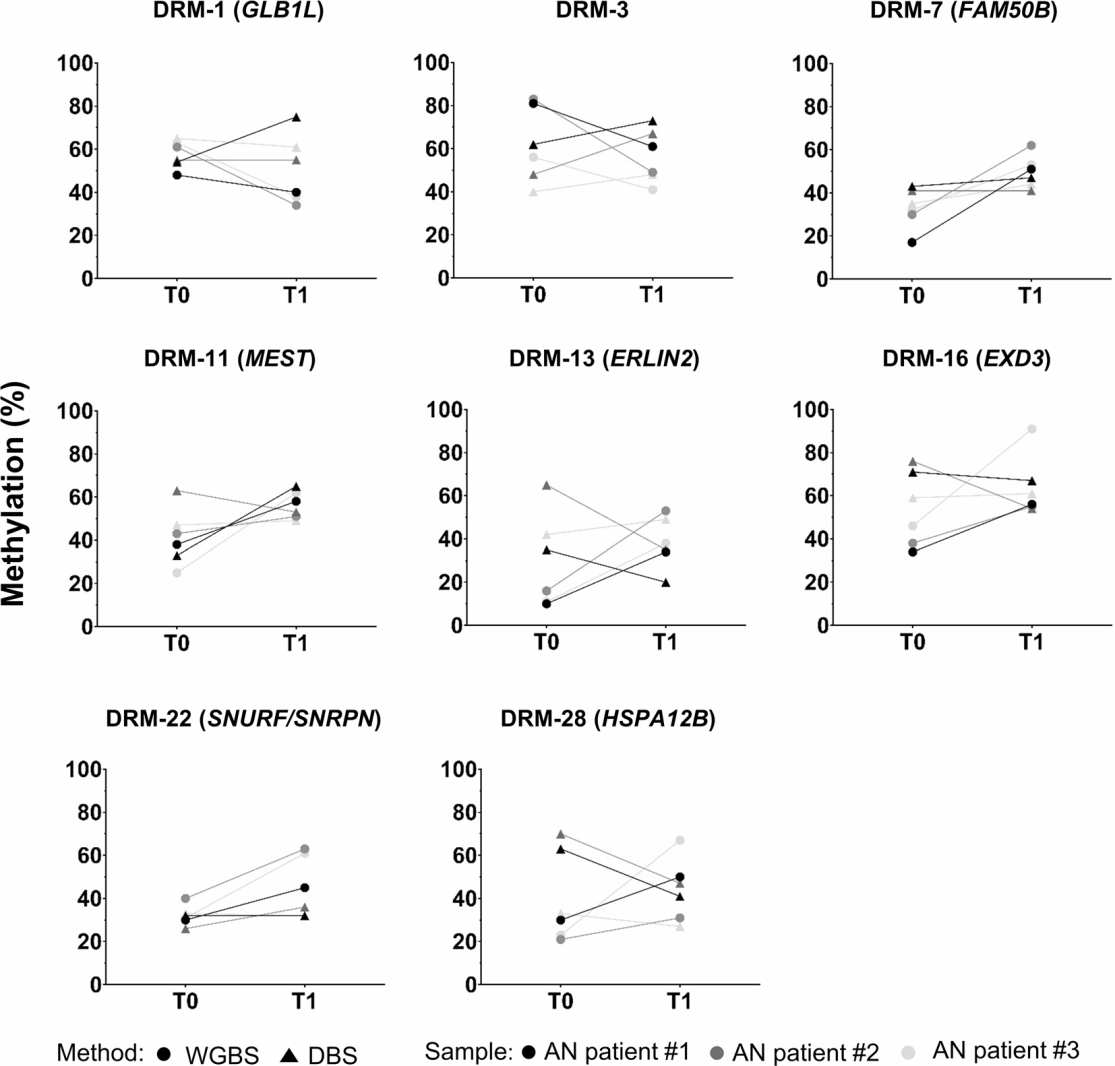
Technical replication of eight WGBS-derived target regions from three female patient with anorexia nervosa (AN) for pre- (T0) and post-treatment induced weight gain (T1) using DBS. DBS, deep bisulfite sequencing; WGBS, whole-genome bisulfite sequencing.

Based on the DMR identification (using the decreased threshold), we aimed to technically replicate eight regions (four detected by both DMR calling tools, and four detected by camel; see Supplementary Tables 4 and 5) using deep bisulfite sequencing (DBS) in the same three individuals (pre- and post- weight gain, see Table 2). These regions comprised: DMR-1 within Galactosidase Beta 1 Like (*GLB1L*), intergenic DMR-3, DMR-7 within Family With Sequence Similarity 50 Member B (*FAM50B*), DMR-11 within Mesoderm Specific Transcript (*MEST*), DMR-13 within ER Lipid Raft Associated 2 (*ERLIN2*), DMR-16 within Exonuclease 3’-5’ Domain Containing 3 (*EXD3*), DMR-22 within *SNRPN* Upstream Reading Frame/Small Nuclear Ribonucleoprotein Polypeptide N (*SNURF/SNRPN*) and DMR-28 within Heat Shock Protein Family A (*Hsp70*) Member 12B (*HSPA12B*; see Supplementary Table 4). All CpG sites contained in the DMRs were included in the amplicons of the corresponding polymerase chain reactions (PCRs; see Supplementary Table 1). None of the methylation differences identified by WGBS could be confirmed in any of the investigated regions (see Fig. 2).

## Discussion

Evidence for effects of nutrition on the methylome have emerged (e.g.^6–8^). Yet, the impact of DNA methylation on AN and related symptoms like starvation remain ambiguous^10^. We have previously performed a high-throughput DNA methylation analysis (EWAS) and detected some CpG sites differentially methylated in AN with one locus at *NR1H3*^21^ exhibiting deviating methylation directions compared to an earlier study^20^. Consequently, we aimed to replicate our previous findings^21^. In our replication approach for *NR1H3*, despite a larger sample size, we did not observe evidence for methylation differences between patients with AN and healthy-lean female controls at the analysed CpG sites at *NR1H3.* This contrasts with previous reports from other groups describing a hypermethylation at this site^20,23^. Unlike our former study, we were unable to correct for cell type compositions, as EWAS data were not available for all participants. Without this correction, there is also no evidence for deviations in the data sets of the previous, here re-analysed EWAS study^21^. Summarising, we were unable to replicate methylation differences at *NR1H3*, regardless of the methylation direction previously reported^20,21^.

Subsequently, we analysed weight gain associated DMRs in three patients with AN receiving in-patient treatment. We assumed that, if rapid increases in body weight (within weeks) are associated with methylation changes, the effect of a significant weight gain between admission and discharge on methylation changes should be large and, consequently, detectable in such patients with AN. By comparing DNA methylation patterns in the same individuals pre- and post-weight gain, differences based on genetic variation can be ruled out. However, no evidence for methylation differences were observed, and DMRs detected at a lower than standard thresholds could not be technically confirmed. Generally, our study is underpowered to detect subtle methylation differences, such as methylation changes present in only a few CD14^+^ cells. However, we aimed to detect large differences as previously suggested^36^. While we were unable to confirm our hypothesis that the pronounced weight gain during in-patient treatment heavily alters the DNA methylation, it remains possible that such changes may only become evident after a longer period of time.

Other studies trying to replicate previous findings revealed similar outcomes. For example, one study failed to replicate^18^ the previously reported overall global hypermethylation found on a CpG site at *HDAC4*^20^.Yet, this replication attempt might be hampered by the use of different initial source tissues^18^. In a different study^31^, genome-wide methylation of monozygotic twins discordant for AN were analysed and nine CpG sites at genes were detected. Subsequently, the authors were able to validate their results at least partially in non-familial AN samples and controls applying rank regression and beta regression modelling (two and six prior detected CpG sites, respectively).

In general, many studies have investigated candidate-gene approaches (e.g.^11–19^) and some investigated genome-wide methylation pattern (e.g.^20,21,31^). However, it remains uncertain whether the detected methylation changes and differences are due to AN, its related starvation or due to a varying cell type composition. This is particularly evident, as it is known that cell type composition varies between controls and patients with AN as well as between patients with AN before and after treatment^37^. For instance, it was found that patients with AN had reduced levels of lymphocytes and B-cells after discharge following a six-week treatment in comparison to healthy individuals^37^. Of note, blood is not a primary tissue for AN. Yet, it is easily accessible, can be obtained with minimal invasiveness during routine clinical procedures and allows for sampling of large number of individuals. In fact, primary tissues such as brain or adipose tissue are not ethically feasible to obtain from patients and particularly from controls. In our weight gain-associated DMR identification approach, we decided to use a homogenous cell type (CD14^+^ cells). Nevertheless, different results might be obtained if different cell types would have been chosen (e.g. T-cells, NK-cells, etc.) as DNA methylation is highly cell type-specific^25,26^. Further, we have exclusively analysed three patients with AN. DNA methylation might be highly variable between individuals and thus more subjects would be needed to detect subtle common effects.

It is remarkable that we could not observe evidence for an impact on the methylome by the pronounced weight gain during in-patient treatment. This might be due to the fact that cell type-specific methylation patterns are highly stable. This stability can probably be attributed to stem or progenitor cells. We previously showed differences in metabolic profiles in patients with AN compared to controls and in patients with AN between acute stage of starvation and after a short-term weight recovery. A substantial number of metabolite levels did not return to normal after short term recovery^38^. Yet, after a prolonged period of time, the metabolite levels resembled those of healthy controls^39^. Hence, one might hypothesise that weight gain could exert an effect on the methylome, albeit at a later time point. Putatively, additional factors like the nutritional status^24^ might also be implicated in methylation changes.

Differences in demographic characteristics such as age, weight and BMI were observed between the included study groups (see Table 3). However, these differences are inherently linked to the clinical phenotype of AN, which is typically characterized by markedly reduced body weight and a higher prevalence among adolescent females^2–4^. As such, strict matching on these parameters may not be clinically representative and could obscure relevant disease-specific features. Yet, age is a well-established modulator of DNA methylation, with promoter-associated CpG sites often exhibiting hypermethylation and other genomic regions showing hypomethylation over time^40–43^. Longitudinal data reported a median rate of methylation change of approximately 0.18% per year^44^, indicating rather subtle age-related effects. Notably, the inter-individual variability in methylation patterns can be of similar magnitude to age-related changes^44,45^. Additionally, lifestyle factors such as physical activity, diet and stress have been shown to influence DNA methylation itself as well as the pace of epigenetic aging^42,45,46^. Even among monozygotic twins, DNA methylation patterns become increasingly divergent over time, particularly when exposed to distinct environmental influences^43^. Thus, epigenetic aging and dynamics are governed by a complex interplay of various determinants. While we cannot entirely exclude the possibility that differences in age, weight and BMI may introduce a certain degree of confounding, these variables represent only a subset of the full spectrum of factors influencing the epigenome. In fact, they are closely linked to the clinical manifestation of AN and should thus be considered group-specific characteristics.

**Table 3:** Phenotypic characteristics of the study (sub-) groups used in the replication analyses.

Characteristic		Female patients with AN			Healthy-lean females			*p*-value for group comparison		
		Total	Independent	Non-independent	Total	Independent	Non-independent	Total	Independent	Non-independent
N		198	189	9	87	67	20	NA	NA	NA
Age, in years	Median	18.73	19.52	16.27	23.97	24.97	22.13	1.5 × 10^–5^	4.1 × 10^–5^	8.0 × 10^–7^
	(Q1,Q3)	(15.17, 27.94)	(15.23, 28.52)	(14.68, 17.11)	(22.23, 26.58)	(23.24, 28.02)	(20.67, 22.52)			
Body weight, in kg	Median	44.00	44.40	36.80	51.30	51.30	51.05	3.9 × 10^–13^	2.4 × 10^–10^	2.4 × 10^–6^
	(Q1,Q3)	(40.30, 49.23)	(40.85, 49.95)	(33.30, 40.10)	(48.50, 53.70)	(48.50, 53.80)	(47.28, 52.58)			
BMI, in kg/m^2^	Median	16.36	16.44	13.67	17.57	17.73	17.18	4.7 × 10^–8^	1.3 × 10^–7^	4.0 × 10^–7^
	(Q1,Q3)	(14.84, 17.70)	(15.20, 17.75)	(13.10, 14.72)	(17.21, 18.05)	(17.33, 18.18)	(16.44, 17.41)			

All individuals included in the study were female. Furthermore, the two-sided *p*-values from the Mann–Whitney U test for comparing female patients with AN versus healthy lean females are provided. AN, anorexia nervosa; BMI, body mass index; N, number of individuals; NA, not applicable; Q1, first quartile; Q3, third quartile.

In sum, we could not replicate findings of our previous EWAS approach^21^ and studies by others^20^. Furthermore, we were unable to detect weight gain associated methylation patterns.

## Materials and methods

### Samples: definition and characteristics

Replication study group: The data set for the replication analyses of the *NR1H3* gene locus comprised 285 females in total (see Table 3). Among them, 198 females (69.47%) were previously diagnosed with AN, while 87 healthy-lean females (30.53%) had no diagnosis of AN or any other eating disorder and thus acted as controls. All participants were recruited in Germany. Of the included participants, 29 females (9 with AN, 20 without AN) were already analysed in the high-throughput DNA methylation analyses^21^. Consequently, we additionally defined the subgroup of females that were not included in the previous work (“independent subgroup”) and of females included in the previous work (“non-independent subgroup”). To compare selected patient characteristics, we applied the non-parametric Mann–Whitney U test (using IBM SPSS Statistics 29.0.0 for Windows) and reported the two-sided p-values. The phenotypic characteristics of the study groups are depicted in Table 3. Patients with AN and controls differed in all reported parameters.

DMR identification cohort: For the genome-wide identification of DMRs, three female patients with AN receiving in-patient treatment at the Department of Child and Adolescent Psychiatry, Psychosomatics and Psychotherapy at the University Hospital Essen were included. Inclusion criteria were diagnosis of AN according to DSM-IV-TR^47^, female sex and German ancestry (characteristics: see Table 2). AN diagnosis was confirmed via a clinical examination and a semi-structured interview (Kiddie Schedule for Affective Disorders and Schizophrenia; K-SADS)^48^. Adolescent patients with AN participated in a multimodal multidisciplinary treatment program^49^ based on dialectical behavioural therapy for adolescents that included weight restoration, individual nutritional counselling, individual therapy (twice a week), skill-groups, individual skill training (twice a week), family sessions (fortnightly), and a group psychoeducation program for parents (monthly). During weight restoration, patients received nutritional counselling and meal support training. Therapy goals were a weight gain of at least 500 g per week and a minimum target weight in the range of the 10-20^th^ age-adjusted BMI percentile.

The complete study was approved by the local ethics committee of the Faculty of Medicine at the University Hospital Essen (No. 06-3212 amendment of November 2016). Patients and in case of minors their parents gave written informed consent. This study was conducted in accordance to the *Declaration of Helsinki*^50^*.*

### Replication analyses of the *NR1H3* gene locus

For the statistical evaluation of the replication, we used two analysis sets. The main analysis was performed only with females not included in the original work^21^ to ensure an independent replication. A sensitivity analysis was conducted in the complete sample, i.e. including females from both the independent and the non-independent subgroup. These two analysis sets are subsequently called “independent sample” (containing only females from the independent subgroup) and “non-independent sample” (complete sample).

Within the replication study group, blood was drawn from each participant and DNA was isolated from whole blood using a salting out approach^51^. DNA was bisulfite-modified using the EZ DNA Methylation-Gold Kit (Zymo Research, Orange, CA, USA) according to the manufacturer’s instructions. To eventually allow a pooled sequencing approach, a two-step PCR (Veriti 96-well Thermal Cycler, Applied Biosystems, Foster City, CA, USA) was conducted. The first PCR was performed using *NR1H3*-specific primers with tags at the 5’ end (see Supplementary Table 1). Subsequently, the PCR products were used in a second PCR applying primers with forward and reverse adapters complementary to the tags attached in the first PCR round (see Supplementary Table 2). The usage of unique combinations of forward and reverse adapters allowed an unambiguous assignment of each sample despite a pooled sequencing. PCR products of the second PCR were subsequently purified using the QIAquick 96 PCR Purification Kit (QIAGEN, Venlo, Netherlands). Library preparation and sequencing on the HiSeq2500 Illumina sequencer was performed at the BioChip Lab of the Center of Medical Biotechnology (ZMB) of the University of Duisburg-Essen. Raw sequencing reads were analysed using the amplikyzer2^52^ which enables demultiplexing of the data, creation of FASTQ files and provides the output of DNA methylation values in percent for each CpG.

To investigate the association between the female’s AN status (with AN, healthy-lean controls without AN) and DNA methylation, a robust linear regression modelling was applied for each CpG site. Each model comprised the site-specific DNA methylation value as a dependent variable and the indicator for AN diagnosis (1: AN; 0: no AN) as an independent variable. Due to the candidate CpG site approach in this study, an adjustment for different cell type compositions was not possible. For the robust linear model, MM estimation was applied and the Tukey bi-square function (with the tuning constant c = 4.685) for observation weighting was used. We provide the estimated DNA methylation difference (regression coefficient) together with the 95% confidence interval. A two-sided significance level of 0.05 was used and was not adjusted for multiple comparisons. Analyses were performed with R (version 3.5.1) and the R package MASS (v.7.3–51.4).

### Identification of weight gain associated DMRs

In the DMR identification cohort, whole blood was drawn at admission and discharge. Given the cell type specificity of DNA methylation^25,26,33^, this analysis focused on one homogenous cell population, namely the CD14^+^ monocytes. Monocytes have been demonstrated to play a pivotal role in inflammatory responses^53^, and patients with AN who are acutely ill exhibit a dysregulated inflammatory status^54^. Furthermore, CD14^+^ monocytes have been utilised in epigenetic studies of comorbid psychiatric disorders, such as major depressive disorder^55^, and metabolic conditions, such as the metabolic syndrome^56^, which constitute the opposite end of the BMI spectrum. Therefore, patients’ blood was used to immunomagnetically purify DNA of CD14^+^ monocytes using the Whole-Blood column kit with the StraightFrom® Whole Blood CD14 MicroBeads in humans (Miltenyi Biotec; Bergisch Gladbach, Germany) according to the manufacturer’s instructions. The isolated DNA was bisulfite-modified with the EZ DNA Methylation-Gold Kit (Zymo Research, Orange, CA, USA). The samples were sent for library preparation and subsequent WGBS to the Genomic and Proteomics Core Facility of the German Cancer Research Center (DKFZ) in Heidelberg (Germany). The sequencing was performed on the HiSeqX platform (Illumina, San Diego, CA, USA) with one sample per lane. The resulting read data was processed as previously described^36,57,58^. In brief, reads were aligned against the hg19 reference genome built hg19 using bwa-meth (v0.2.0)^59^ and subsequently sorted using samtools (v1.3.1)^60^. After deduplicating the reads with sambamba (v0.6.0)^61^, quality control applying qualimap (v2.2.2)^62^ was performed. Basic statistics related to the process success are presented in Supplementary Table 1.

For genome-wide DMR calling, we utilized camel^63^ and metilene^64^ following their respective manuals. In the camel analysis, DNA methylation levels were initially called by camel itself, and DMRs were identified based on DNA methylation differences, using a threshold of four CpG sites and a minimum difference of 0.3. As no DMRs were identified at this threshold, we adjusted the parameters to a minimum DNA methylation difference of 0.2 and increased the stringency to a minimum of 10 CpG sites. Additionally, metilene was employed to call DMRs using a significance threshold of q < 0.05. Following DMR identification, we applied PCA using the Python scikit-learn library^65^ to visualize differences of methylation patterns across sample groups. For the PCA, we included CpG loci that had a minimum coverage of 10 reads in all analysed samples and a minimum mapping quality score of 30. Furthermore, hierarchical clustering with a cityblock (Manhattan) metric was performed using scikit-learn on the 1,000 CpG sites with the highest standard deviation across all samples to further explore differences between samples. CpG SNPs were excluded from this analysis^66^.

To validate the WGBS-derived DMRs, a DBS approach was performed for eight DMRs (four detected by both DMR calling tools, and four detected by camel only) in the DMR identification cohort. We applied a two-step PCR similar to the one used for the *NR1H3* gene locus applying DMR-specific primers (see Supplementary Table 2). PCR products were purified using the QIAquick 96 PCR Purification Kit (QIAGEN, Venlo, Netherlands). The DBS was performed at the Genomics and Transcriptomics Facility at the University Hospital Essen (Germany). We visually analysed whether the studied DMRs replicated technically using the amplikyzer software^52^.

## Electronic supplementary material

Below is the link to the electronic supplementary material.

Supplementary Material 1

## Data Availability

The original contributions presented in the study are publicly available. This data can be found here: https://www.ebi.ac.uk/ena/browser/view/PRJEB38906.
