# Supplementary material for: No indications of weight gain associated DNA methylation changes in patients with anorexia nervosa
Source: Sci Rep. 2025 Aug 7;15:28870. doi: 10.1038/s41598-025-12592-5 (PMC12331989; doi:10.1038/s41598-025-12592-5)
Supplement: Supplementary file 1 — Supplementary Material 1 [file 41598_2025_12592_MOESM1_ESM.docx]

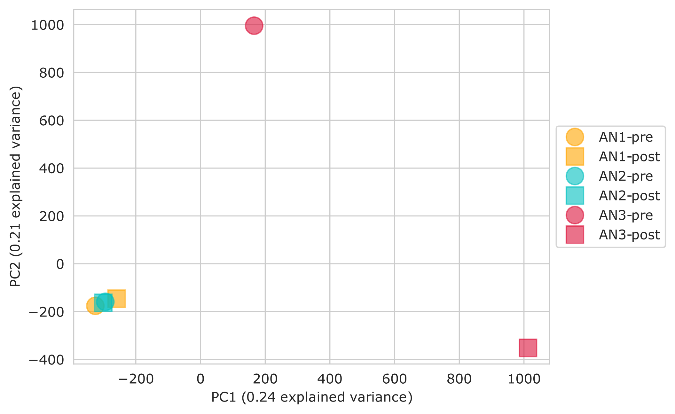


**Supplementary Figure 1: Principal component analysis (PCA) of the six anorexia nervosa (AN) datasets of three female patients with AN receiving in-patient treatment.** For all females, data was collected pre- and post-treatment. CpG sites with a minimum coverage of 10 reads in all samples and a minimum mapping quality of 30 were included in this PCA. In total, 23 million CpGs were included. The first two principal components (PC1, PC2) are presented.

**Supplementary Table 1: Basic statistics of the whole-genome bisulfite sequencing (WGBS) libraries.**

| **Patient** | **Sample time point** | **Conversion** | **Aligned** | **Duplication rate** | **Coverage** | **Mean methylation** |
| --- | --- | --- | --- | --- | --- | --- |
| #1: AS_222872-LR-34031 | pre-treatment | 1.00 | 1.00 | 0.19 | 21.15 | 0.76 |
| #1: AS-222873-LR-34032 | post-treatment | 1.00 | 1.00 | 0.18 | 22.54 | 0.76 |
| #2: AS-222875-LR-34033 | pre-treatment | 1.00 | 1.00 | 0.17 | 21.13 | 0.76 |
| #2: AS-222876-LR-34034 | post-treatment | 1.00 | 1.00 | 0.15 | 21.17 | 0.76 |
| #3: AS-222877-LR-34035 | pre-treatment | 1.00 | 1.00 | 0.20 | 18.75 | 0.75 |
| #3: AS-241990-LR-35191 | post-treatment | 1.00 | 1.00 | 0.17 | 18.00 | 0.74 |

**Supplementary Table 2:** **Primer sequences used.** PCRs were performed as described in Leitão et al. (2018) in a two-step approach. Sequences shown in red represent tag sequences used as a template for the second round of PCR. Amplicon length refers to the amplicon length without the given tag sequence.

| **DMR / Gene** | **Primer** | **Sequence (5´🡪 3´)** | **Amplicon length (bp)** | **Number of analyzed CpG sites** |
| --- | --- | --- | --- | --- |
| - / NR1H3 | forward | CTTGCTTCCTGGCACGAGAGGTGTTTTGTTAGAAGAATAGATT | 319 | 15 |
|  | reverse | CAGGAAACAGCTATGACATAAACCAAAACTACCCAAAAAAAA |  |  |
| DMR-1 / *GLB1L* | forward | CTTGCTTCCTGGCACGAGGAAAGTAGTGTAGGTTGTTAGA | 354 | 22 |
|  | reverse | CAGGAAACAGCTATGACAACTCCCAAAAAACTATCC |  |  |
| DMR-3 / - | forward | CTTGCTTCCTGGCACGAGAAAAGGTTTTAATGTAGAATAAAAAT | 230 | 28 |
|  | reverse | CAGGAAACAGCTATGACTAACAAAAAAATTATACAAACTCC |  |  |
| DMR-7 / *FAM50B* | forward | CTTGCTTCCTGGCACGAGGAGGYGTAGAGTTGAGTATTTTT | 246 | 19 |
|  | reverse | CAGGAAACAGCTATGACAAAACCTCTTATCCACCTA |  |  |
| DMR-11 / *MEST* | forward | CTTGCTTCCTGGCACGAGTGTAGGATTTTTAGAATTTTAGT | 309 | 22 |
|  | reverse | CAGGAAACAGCTATGACCAACATAACAATTTAATCACATC |  |  |
| DMR-13 / *ERLIN2* | forward | CTTGCTTCCTGGCACGAGTAGTAGTATATATGGAGGGGTTTTT | 230 | 16 |
|  | reverse | CAGGAAACAGCTATGACTAAAAATATTAATAAATACCATTTAAATTA |  |  |
| DMR-16 / *EXD3* | forward | CTTGCTTCCTGGCACGAGGGGTTTTGGTAGTTTTTTTT | 357 | 44 |
|  | reverse | CAGGAAACAGCTATGACACTCCTCAAATCCTCAAACTCTATC |  |  |
| DMR-22 / *SNURF*/*SNRPN* | forward | CTTGCTTCCTGGCACGAGGGGATTAGTGTATAGGGATTTTAGG | 302 | 19 |
|  | reverse | CAGGAAACAGCTATGACCTTCCCCCTACCTCCCAA |  |  |
| DMR-28 / *HSPA12B* | forward | CTTGCTTCCTGGCACGAGGTTTAGTTTYGAGTTTGAGTT | 354 | 41 |
|  | reverse | CAGGAAACAGCTATGACCAATCTCTAAATATATCCCCACC |  |  |

**Supplementary Table 3: Adapter sequences applied in the second round of PCR.** Sequences representing the tags are shown in red.

| **Adapter name** | **Sequence (5‘ 🡪 3‘)** |
| --- | --- |
| i5 S510 Illum Ftag | AATGATACGGCGACCACCGAGATCTACACCGTCTAATACACTCTTTCCCTACACGACGCTCTTCCGATCTCTCTCT  CTTGCTTCCTGGCACGAG |
| i5 S511 Illum Ftag | AATGATACGGCGACCACCGAGATCTACACTCTCTCCGACACTCTTTCCCTACACGACGCTCTTCCGATCTTATCCT  CTTGCTTCCTGGCACGAG |
| i5 S513 Illum Ftag | AATGATACGGCGACCACCGAGATCTACACTCGACTAGACACTCTTTCCCTACACGACGCTCTTCCGATCTAGAGTA  CTTGCTTCCTGGCACGAG |
| i5 S515 Illum Ftag | AATGATACGGCGACCACCGAGATCTACACTTCTAGCTACACTCTTTCCCTACACGACGCTCTTCCGATCTGTAAGG  CTTGCTTCCTGGCACGAG |
| i5 S516 Illum Ftag | AATGATACGGCGACCACCGAGATCTACACCCTAGAGTACACTCTTTCCCTACACGACGCTCTTCCGATCTACTGCA  CTTGCTTCCTGGCACGAG |
| i5 S518 Illum Ftag | AATGATACGGCGACCACCGAGATCTACACCTATTAAGACACTCTTTCCCTACACGACGCTCTTCCGATCTCTAAGC  CTTGCTTCCTGGCACGAG |
| i5 S521 Illum Ftag | AATGATACGGCGACCACCGAGATCTACACGAGCCTTAACACTCTTTCCCTACACGACGCTCTTCCGATCTTATAGC  CTTGCTTCCTGGCACGAG |
| i5 S522 Illum Ftag | AATGATACGGCGACCACCGAGATCTACACTTATGCGAACACTCTTTCCCTACACGACGCTCTTCCGATCTATAGAG  CTTGCTTCCTGGCACGAG |
| i7 N714 Illum Rtag | CAAGCAGAAGACGGCATACGAGATTCATGAGCGTGACTGGAGTTCAGACGTGTGCTCTTCCGATCT  CAGGAAACAGCTATGAC |
| i7 N715 Illum Rtag | CAAGCAGAAGACGGCATACGAGATCCTGAGATGTGACTGGAGTTCAGACGTGTGCTCTTCCGATCT  CAGGAAACAGCTATGAC |
| i7 N716 Illum Rtag | CAAGCAGAAGACGGCATACGAGATTAGCGAGTGTGACTGGAGTTCAGACGTGTGCTCTTCCGATCT  CAGGAAACAGCTATGAC |
| i7 N718 Illum Rtag | CAAGCAGAAGACGGCATACGAGATGTAGCTCCGTGACTGGAGTTCAGACGTGTGCTCTTCCGATCT  CAGGAAACAGCTATGAC |
| i7 N719 Illum Rtag | CAAGCAGAAGACGGCATACGAGATTACTACGCGTGACTGGAGTTCAGACGTGTGCTCTTCCGATCT  CAGGAAACAGCTATGAC |
| i7 N720 Illum Rtag | CAAGCAGAAGACGGCATACGAGATAGGCTCCGGTGACTGGAGTTCAGACGTGTGCTCTTCCGATCT  CAGGAAACAGCTATGAC |
| i7 N721 Illum Rtag | CAAGCAGAAGACGGCATACGAGATGCAGCGTAGTGACTGGAGTTCAGACGTGTGCTCTTCCGATCT  CAGGAAACAGCTATGAC |
| i7 N722 Illum Rtag | CAAGCAGAAGACGGCATACGAGATCTGCGCATGTGACTGGAGTTCAGACGTGTGCTCTTCCGATCT  CAGGAAACAGCTATGAC |
| i7 N723 Illum Rtag | CAAGCAGAAGACGGCATACGAGATGAGCGCTAGTGACTGGAGTTCAGACGTGTGCTCTTCCGATCT  CAGGAAACAGCTATGAC |
| i7 N724 Illum Rtag | CAAGCAGAAGACGGCATACGAGATCGCTCAGTGTGACTGGAGTTCAGACGTGTGCTCTTCCGATCT  CAGGAAACAGCTATGAC |
| i7 N726 Illum Rtag | CAAGCAGAAGACGGCATACGAGATGTCTTAGGGTGACTGGAGTTCAGACGTGTGCTCTTCCGATCT  CAGGAAACAGCTATGAC |
| i7 N727 Illum Rtag | CAAGCAGAAGACGGCATACGAGATACTGATCGGTGACTGGAGTTCAGACGTGTGCTCTTCCGATCT  CAGGAAACAGCTATGAC |

**Supplementary Table 4: Differentially methylated regions (DMR) in the anorexia nervosa (AN) cohort identified with camel.** All numbers are part of the camel output. X indicates, whether the DMR region met the item of the respective column.

| **DMR**  **number** | **Chromosome** | **Start (bp)** | **Stop (bp)** | **Number of CpG**  **sites** | **Mean**  **methylation**  **difference** | **Gene** | **CpG island** | **Identified with**  **camel and**  **metilene** | **Replicated by deep bisulfite sequencing** |
| --- | --- | --- | --- | --- | --- | --- | --- | --- | --- |
| 1 | 2 | 220108022 | 220108240 | 15 | -0.20 | *GLB1L* | X | X | X |
| 2 | 3 | 122631656 | 122631863 | 19 | 0.20 | *SEMA5B* | X |  |  |
| 3 | 3 | 96495654 | 96495787 | 25 | -0.24 | *-* | X | X | X |
| 4 | 3 | 128372270 | 128372392 | 12 | -0.22 | *RPN1* |  |  |  |
| 5 | 5 | 23951406 | 23951556 | 23 | 0.21 | *-* |  |  |  |
| 6 | 5 | 131607580 | 131607728 | 15 | 0.23 | *PDLIM4* | X |  |  |
| 7 | 6 | 3850030 | 3850198 | 16 | 0.25 | *FAM50B* | X | X | X |
| 8 | 6 | 103780330 | 103780520 | 12 | 0.24 | *-* |  |  |  |
| 9 | 6 | 144329661 | 144329803 | 14 | 0.21 | *PLAGL1* | X | X |  |
| 10 | 7 | 50850481 | 50850557 | 10 | 0.21 | *GRB10* | X |  |  |
| 11 | 7 | 130132831 | 130132968 | 14 | 0.21 | *MEST* | X | X | X |
| 12 | 7 | 138349196 | 138349306 | 12 | 0.21 | *SVOPL* | X | X |  |
| 13 | 8 | 37605479 | 37605612 | 13 | 0.27 | *ERLIN2* |  | X | X |
| 14 | 8 | 145577563 | 145577699 | 12 | -0.21 | *TMEM249* | X |  |  |
| 15 | 9 | 121571655 | 121571834 | 22 | 0.28 | *-* |  |  |  |
| 16 | 9 | 140312005 | 140312139 | 22 | 0.28 | *EXD3* | X | X | X |
| 17 | 9 | 140311650 | 140311755 | 14 | -0.26 | *EXD3* | X | X |  |
| 18 | 10 | 2543763 | 2543863 | 10 | 0.24 | *-* | X |  |  |
| 19 | 10 | 134312364 | 134312602 | 11 | 0.23 | *-* |  |  |  |
| 20 | 12 | 297564 | 297660 | 13 | 0.20 | *-* |  |  |  |
| 21 | 14 | 104394643 | 104394730 | 18 | 0.25 | *-* |  |  |  |
| 22 | 15 | 25200653 | 25200867 | 17 | 0.21 | *SNURF/SNRPN* | X | X | X |
| 23 | 16 | 60558 | 60623 | 18 | -0.24 | *-* |  | X |  |
| 24 | 19 | 1423673 | 1423847 | 12 | 0.22 | *DAZAP1* | X |  |  |
| 25 | 19 | 50435883 | 50436183 | 13 | 0.22 | *ATF5* |  |  |  |
| 26 | 19 | 54927810 | 54928046 | 22 | 0.21 | *TTYH1* | X |  |  |
| 27 | 20 | 3732270 | 3732372 | 17 | 0.23 | *HSPA12B* | X |  |  |
| 28 | 20 | 3732712 | 3732826 | 19 | 0.27 | *HSPA12B* | X | X | X |
| 29 | 20 | 57427046 | 57427278 | 12 | 0.21 | *GNAS* | X |  |  |
| 30 | 20 | 57416457 | 57416590 | 14 | -0.23 | *GNAS/GNAS-AS1* | X |  |  |
| 31 | X | 47509923 | 47510197 | 27 | 0.21 | *ELK1* | X | X |  |
| 32 | X | 128657066 | 128657256 | 16 | 0.28 | *SMARCA1* | X |  |  |
| 33 | X | 153599077 | 153599242 | 22 | 0.24 | *FLNA* | X |  |  |
| 34 | X | 103411321 | 103411411 | 12 | -0.21 | *FAM199X* | X |  |  |
| 35 | X | 119444838 | 119444969 | 13 | -0.21 | *TMEM255A* | X |  |  |

**Supplementary Table 5:** **Differentially methylated regions (DMR) in the anorexia nervosa (AN) cohort identified with metilene.** All numbers are part of the metilene output. X indicates, whether the DMR region met the item of the respective column. 2D KS test: 2-dimensional Kolmogorov-Smirnov test, MWU test: Mann-Whitney U test

| **Chromo-some** | **Start (bp)** | **Stop (bp)** | **q-value** | **Mean methylation difference** | **Number of CpG sites** | **p-value of**  **MWU test** | **p-value of**  **2D KS test** | **Pre-treatment mean methylation level** | **Post-treatment mean methylation level** | **Gene** | **CpG island** | **Identified with camel and metilene** | **Replicated by deep bisulfite sequencing** |
| --- | --- | --- | --- | --- | --- | --- | --- | --- | --- | --- | --- | --- | --- |
| 1 | 108023278 | 108023429 | 4.1E-03 | 8.08 | 17 | 4.4E-11 | 4.7E-10 | 18.22 | 10.14 | *NTNG1* | X |  |  |
| 2 | 22018022 | 220108240 | 9.0E-01 | 20.02 | 9 | 2.7E -10 | 1.0E-07 | 57.36 | 37.33 | *GLB1L* | X | X | X |
| 3 | 96495630 | 96495787 | 2.6E-02 | 23.22 | 26 | 8.7E-14 | 2.9E-09 | 73.32 | 50.10 | - | X | X | X |
| 5 | 124071060 | 124071119 | 1.3E-02 | 4.94 | 11 | 2.0E-09 | 1.5E-09 | 5.48 | 0.55 | *ZNF608* |  |  |  |
| 5 | 179740839 | 179741042 | 3.5E-03 | -19.86 | 23 | 4.3E-14 | 4.0E-10 | 35.33 | 55.19 | *GFPT2* | X |  |  |
| 6 | 3850038 | 3850119 | 2.6E-01 | -28.81 | 9 | 1.2E-08 | 3.0E-08 | 26.30 | 55.11 | *FAM50B* | X | X | X |
| 6 | 17016240 | 17016374 | 6.6E-06 | 9.00 | 19 | 1.5E-13 | 7.6E-13 | 9.86 | 0.86 | - |  |  |  |
| 6 | 117868983 | 117869249 | 9.9E-03 | 5.88 | 23 | 2.6E-11 | 1.1E-09 | 7.22 | 1.33 | *DCBLD1* | X |  |  |
| 6 | 144329638 | 144329830 | 1.8E-03 | -19.49 | 17 | 7.1E-12 | 2.1E-10 | 32.82 | 52.31 | *PLAGL1* | X | X |  |
| 7 | 50849910 | 50849969 | 2.6E-02 | -16.70 | 9 | 5.0E-10 | 2.9E-09 | 30.00 | 46.70 | *GRB10* | X |  |  |
| 7 | 105596474 | 105596577 | 2.1E-03 | -22.90 | 14 | 4.7E-13 | 2.4E-10 | 60.05 | 82.95 | - |  |  |  |
| 7 | 130132857 | 130132968 | 1.9E-02 | -21.81 | 12 | 3.3E-11 | 2.2E-09 | 35.25 | 57.06 | *MEST* | X | X | X |
| 7 | 138349215 | 138349321 | 3.0E-03 | -21.06 | 11 | 1.6E-11 | 3.4E-10 | 16.73 | 37.79 | *SVOPL* | X | X |  |
| 8 | 37605479 | 37605612 | 3.9E-05 | -28.76 | 11 | 5.5E-12 | 4.5E-12 | 12.79 | 41.55 | *ERLIN2* |  | X | X |
| 8 | 144358840 | 144358981 | 7.0E-04 | -7.67 | 17 | 4.9E-14 | 7.6E-11 | 2.75 | 10.41 | *GLI4* | X |  |  |
| 9 | 121571655 | 121571834 | 1.0E-03 | -28.48 | 21 | 4.3E-14 | 1.2E-10 | 43.92 | 72.40 | - |  | X |  |
| 9 | 140312005 | 140312139 | 3.7E-07 | -27.61 | 22 | 4.2E-14 | 4.3E-14 | 39.71 | 67.32 | *EXD3* | X | X | X |
| 12 | 54473384 | 54473709 | 1.0E-03 | 12.61 | 17 | 6.8E-12 | 1.1E-10 | 93.69 | 81.08 | *FLJ12825* | X |  |  |
| 12 | 132469818 | 132469983 | 1.3E-03 | 6.15 | 13 | 4.1E-11 | 1.5E-10 | 6.69 | 0.54 | *EP400* |  |  |  |
| 14 | 42582922 | 42583023 | 2.2E-03 | -14.72 | 19 | 1.9E-13 | 2.5E-10 | 81.61 | 96.33 | - |  |  |  |
| 14 | 70700881 | 70701005 | 5.4E-03 | -16.32 | 22 | 1.8E-13 | 6.2E-10 | 75.33 | 91.65 | - | X |  |  |
| 14 | 101291487 | 101292045 | 6.5E-03 | -15.32 | 26 | 2.5E-13 | 7.4E-10 | 35.49 | 50.81 | *MEG3* | X |  |  |
| 15 | 25200648 | 25200867 | 1.6E-01 | -22.22 | 15 | 6.8E-11 | 1.8E-08 | 33.38 | 55.60 | *SNURF/SNRPN* | X | X | X |
| 16 | 60504 | 60682 | 9.3E-05 | 21.84 | 29 | 4.2E-14 | 1.1E-11 | 55.13 | 33.29 | - |  | X |  |
| 16 | 15083956 | 15084164 | 2.1E-02 | -16.32 | 29 | 4.7E-14 | 2.4E-09 | 25.93 | 42.25 | *PDXDC1* | X |  |  |
| 17 | 40822127 | 40822226 | 4.9E-03 | 16.33 | 14 | 1.1E-12 | 5.6E-10 | 18.81 | 2.48 | *PLEKHH3* | X |  |  |
| 19 | 5229443 | 5229639 | 6.5E-06 | 14.81 | 31 | 4.4E-14 | 7.5E-13 | 19.71 | 4.90 | *PTPRS* | X |  |  |
| 19 | 49982111 | 49982229 | 4.6E-02 | 12.08 | 8 | 6.9E-09 | 5.3E-09 | 25.04 | 12.96 | *FLT3LG* |  |  |  |
| 20 | 3732696 | 3732828 | 6.3E-02 | -24.61 | 24 | 1.1E-13 | 7.2E-09 | 24.57 | 49.18 | *HSPA12B* | X | X | X |
| 22 | 29075511 | 29075692 | 4.6E-06 | -13.84 | 25 | 4.6E-14 | 5.3E-13 | 2.48 | 16.32 | *TTC28* | X |  |  |
| X | 46618447 | 46618564 | 4.3E-03 | 24.73 | 17 | 1.0E-13 | 4.9E-10 | 33.02 | 8.29 | *SLC9A7* | X |  |  |
| X | 47509923 | 47510197 | 2.0E-04 | -19.89 | 27 | 4.4E-14 | 2.0E-11 | 21.01 | 40.90 | *ELK1* | X | X |  |
| X | 130930007 | 130930161 | 1.4E-02 | -17.89 | 15 | 1.0E-10 | 1.6E-09 | 23.40 | 41.29 | *FIRRE* | X |  |  |
| X | 153657063 | 153657259 | 4.0E-04 | 21.71 | 28 | 4.1E-14 | 4.4E-11 | 35.60 | 13.88 | *ATP6AP1* | X |  |  |
